# The Complex Adaptive Delta-Modulator in Sliding Mode Theory

**DOI:** 10.3390/e22080814

**Published:** 2020-07-25

**Authors:** Dhafer Almakhles

**Affiliations:** 1 College of Engineering, Communications & Networks Engineering, Prince Sultan University, Riyadh 11586, Saudi Arabia; dalmakhles@psu.edu.sa or rel@psu.edu.sa; 2 Renewable Energy Lab, Prince Sultan University, Riyadh 11586, Saudi Arabia

**Keywords:** adaptive delta-modulator, quasi-sliding mode, two-level quantizer, hitting-time, single-bit, periodicity

## Abstract

In this paper, we consider the stability and various dynamical behaviors of both discrete-time delta modulator (Δ-M) and adaptive Δ-M. The stability constraints and conditions of Δ-M and adaptive Δ-M are derived following the theory of quasi-sliding mode. Furthermore, the periodic behaviors are explored for both the systems with steady-state inputs and certain parameter values. The results derived in this paper are validated using simulated examples which confirms the derived stability conditions and the existence of periodicity.

## 1. Introduction

Over the last few years, the quantizers and modulators have gain popularity due to their important role in many engineering fields including measurement, instrumentation, power converters and analog to digital conversion (ADC) [1,2]. In particular, the binary quantizers including delta modulators (Δ-Ms) and delta sigma modulators (ΔΣ-Ms) are widely utilized in power conversions for their implementation simplicity [3,4], networked and quantized control systems for their low-data rate [5,6], and as typical complex systems for their high nonlinearity, periodicity and robustness at the same time [7,8]. Such systems are often used as coding mechanism in many applications such bio-medical engineering (see [9,10]) due to their low energy consumption and ability in data compression and for reducing the wiring in communication systems where the communication channels are shared by many entities and hardware resources are limited [11,12].

In practical engineering applications, the Δ-M is often implemented either in continuous or discrete-time domain. However, it is important to point out that the selection of the domains, discrete-time (DT) Δ-M and its continuous counterpart, the continuous-time (CT) Δ-M, is often subject to the nature of the interconnection of components and devices in the applications [12]. For instance, using CT Δ-M is fitting in continuous-time systems where the input/output signals are continuous-time. Similarly, in discrete-time systems where the input/output signals are discrete-time, it is proper to utilize DT Δ-M. Further, in some hybrid applications, the Δ-M can be implemented either in analog or digital integrated circuit frameworks depending on the availability of the hardware resources and the technical expertise of the designer.

A typical structure of of both Δ-M and ΔΣ-M consist of a transmitter (encoder) connected to a receiver (decoder) through a binary communication channel. The systems are often classified as dynamic quantizers [12]. As other quantizers, they contain coarsest quantizers (relay components) where high nonlinearity and complex behavior are inherently exhibited [12,13]. Despite the fact that both systems have many resemblances, each one of them has some different features which differentiate them. For example, the output of the transmitter is a binary signal that represents the derivative of the input signal of Δ-M. Thus, Δ-M is also referred to as differential modulator and the demodulation at the receiver side is preceded by an integrator to reconstruct the input signals [14]. However, the output of the transmitter of ΔΣ-M is an over-sampled binary signal that contains the dynamic ranges of the low-frequency part of the input signal (so-called noise shaping feature) [15]. To reduce the quanitization errors inserted by Δ-M and ΔΣ-M systems, the output signals should be processed with decimation filters to generate the quantized counterpart of the input signals. In particular, the conventional Δ-M can roughly be classified as a two-level dynamic quanitizer with fixed step-size (alternately referred here to as fixed Δ-M) whereas Δ-M with adaptive quantizer step-size is alternately referred to as adaptive Δ-M [14]. It is essential to mention that the selection of the gains plays a critical role in the performance and the stability of Δ-M [16,17]. Thus, to alleviate the demerits associated with the standard Δ-M, the adaptive Δ-M attempts to increase the dynamic range of the dynamic range and stability region of the fixed Δ-M [18,19,20].

In the literature on Δ-M, some works analyzed the dynamical behavior of the fixed Δ-M in terms of stability and periodicity. For example, the stability analysis and periodicity of self excited and fixed Δ-M is carried out using sliding mode theory in [6,7,8,16,21], which is often used for the analysis of such chaotic systems with complex behaviors [22,23]. In [24], authors briefly studied the stability of data-driven Δ-M, where the equivalent control-based sliding mode is applied to investigate the stability analysis and the dynamical behavior of the fixed Δ-M. However, it is remarked that the dynamical behavior and stability analysis for the adaptive Δ-M have not been reported in the literature.

Motivated by the above discussion, the presented work investigates inherent dynamical characterizations of data-driven fixed Δ-M and adaptive Δ-M in DT domains. We state the stability conditions, define the periodicity and accurately approximate the reaching time (so-called hitting-step) for both the DT fixed and adaptive Δ-Ms using sliding mode theory. The appropriate quantizer gainfor the fixed Δ-Mis determined using the dynamics of the input signals. With appropriate choices of quantizer gain and parameters of the fixed and adaptive Δ-M, we show that both fixed and adaptive Δ-Mconverge to periodic-2 and periodic-4 orbit in the steady-state, respectively. The analytically established findings in this paper are validated via numerical simulations.

The organization of this paper is thus. In Section 2, we discuss the dynamic properties of the fixed Δ-M in DT domain with more focusing on the periodicity. Then, we study the stability and periodicity of the adaptive Δ-M in DT domain in Section 3. The simulations of both are presented in Section 4. Finally, we conclude and summarize our results in Section 5.

## 2. Fixed Delta Modulator (Fixed Δ-M)

Before proceeding to study the dynamical characterizations of both the fixed and adaptive Δ-Ms, it is of the essence to define the quasi-sliding mode and reaching condition.

**Definition** **1** ([24]). The DT sliding mode trajectory $s_{k}$ is described by so-called quasi-sliding mode in the vicinity ε if $\left|s_{k}\right|\leq \varepsilon$ holds for $k>k_{f}$, in which $k_{f}$ is a positive integer. The domain for the motion of $s_{k}$ is known as the quasi-sliding mode-domain (QSMD). The ε, which bounds the motion of the trajectory $s_{k}$, is known as the quasi-sliding mode-band (QSMB). Moreover, the quasi-sliding mode satisfies the reaching condition for any $k_{r}\leq k_{f}$ if

(1a) $$\begin{array}{ccc} s_{k_{r}}>\varepsilon & \Rightarrow & -\varepsilon <s_{k_{r}+1}<s_{k_{r}} \end{array}$$

(1b) $$\begin{array}{ccc} s_{k_{r}}<-\varepsilon & \Rightarrow & s_{k_{r}}<s_{k_{r}+1}<\varepsilon \end{array}$$

(1c) $$\begin{array}{ccc} \left|s_{k}\right|\leq \varepsilon & \Rightarrow & \left|s_{k+1}\right|\leq \varepsilon \;\;\forall \;k>k_{f} \end{array}$$

in which $k_{r}$ represents the reaching steps.

As per the definition of quasi-sliding mode, there are two possible cases for the motion of s(k),

- Case 1: before s(k) enters the vicinity ($\left|s_{k}\right|>\varepsilon$), represented by (1a) and (1b).
- Case 2: after s(k) enters the the vicinity ($\left|s_{k}\right|\leq \varepsilon$), represented by Equation (1c).

If the trajectory satisfies the reaching conditions (1.a) and (1.b), then it will clearly move from case 1 to case 2, ultimately. The motion of the trajectory in case 1 is called reaching phase and the steps are also called reaching steps ($k_{r}$). In this work, the final step in case 1 is denoted by as ($k_{f}$).

**Figure 1:**
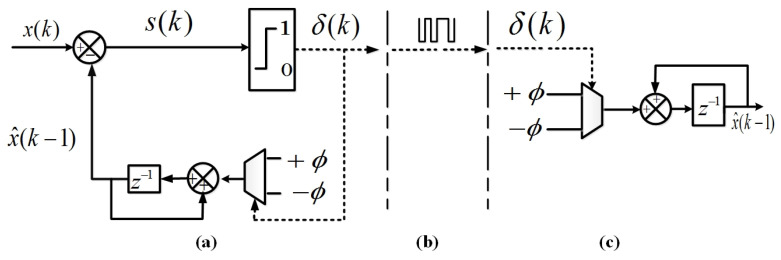
Discrete-time fixed Δ-M (**a**) the encoder of the fixed Δ-M ($E_{\Delta }^{x}$), (**b**) binary communication channel links the encoder to the decoder and (**c**) the decoder of the fixed Δ-M ($D_{\Delta }^{x}$).

The dynamics of the fixed Δ-M depicted in Figure 1 are described by the following equations

(2a) $$\begin{array}{cc} {\hat{x}}_{k+1} & ={\hat{x}}_{k}+\phi \delta_{k}\;\;. \end{array}$$

(2b) $$\begin{array}{cc} s_{k} & =x_{k}-{\hat{x}}_{k} \end{array}$$

where $\delta_{k}=\mathrm{sgn}\left(s_{k}\right)\in \left\{-1,1\right\}$ in which $\mathrm{sgn}(s_{k})=1$ if $s_{k}\geq 0$ and $\mathrm{sgn}(s_{k})=-1$ if $s_{k}<0$. The binary communication channel, which exists between the transmitter (encoder) and the receiver (decoder) carries 1-bit signal defined as:

$${\bar{\delta }}_{k}=\frac{1}{2}\left(1+\delta_{k}\right)\in \left\{0,1\right\}.$$

The notation of the transmitted binary code $\left\{0,1\right\}$ is denoted by ${\bar{\delta }}_{k}$ whereas sgn function that ranges $\left\{-1,1\right\}$ is dented by $\delta_{k}$ (i.e., $\delta_{k}=sgn\left(s_{k}\right)$). The transmitted binary code with sgn function such that ${\bar{\delta }}_{k}=1$ when $\delta_{k}=1$ and ${\bar{\delta }}_{k}=0$ when $\delta_{k}=-1$.

**Lemma** **1.** *Consider* (2), *it follows that*

(3) $$s_{k+1}=\Delta x_{k}+s_{k}-\phi \delta_{k}$$

*where* $\Delta x_{k}=x_{k+1}-x_{k}$.

**Proof.**  Substituting (2b) into (2a) gives

(4) $$\begin{array}{cc} {\hat{x}}_{k+1} & =x_{k}-s_{k}+\phi \;\delta_{k}. \end{array}$$

It follows from (2) and (4) that

$$\begin{array}{cc} s_{k+1} & =x_{k+1}-{\hat{x}}_{k+1}\\ & =x_{k+1}-\left(x_{k}-s_{k}+\phi \delta_{k}\right)\\ & =\Delta x_{k}+s_{k}-\phi \delta_{k}\;. \end{array}$$

 □

### Stability, Periodicity and Estimation of the Hitting-Step for Fixed Δ-M

The following theorem is devoted to derive the stability conditions of the fixed Δ-M.

**Theorem** **1.** *Recall the switching function in* (3). *If*

(5) $$\begin{array}{c} \left|\Delta x_{k}\right|\leq \chi <\phi \; \end{array}$$

*where $\Delta x_{k}=x_{k+1}-x_{k}$ and $\chi \in R_{+}$, then the following holds:*

1. The system trajectory satisfies the reaching conditions of QSM (1) where QSMD is bounded by ε where ε=χ+ϕ.
2. The sliding mode state $s_{k}$ requires at most $k_{f}$ steps to cross the switching manifold $s_{k}=0$, where $k_{r}\leq k_{f}=\left\lfloor m\right\rfloor$ (the notation   denotes the floor operation) and
  (6) $$m=\frac{\left|s_{0}\right|}{\phi -\chi }\;.$$
3. For the special case of an input signal $x_{k}$ which does not change over time i.e., $\Delta x_{k}=0$, the trajectory of (2) converges to a symmetric periodic-2 orbit.

**Proof.**  The proof of the first point of Theorem 1 is divided into two cases (Case-1 and 2) depending on whether $s_{k}>0$ or $s_{k}<0$. For case-1, where $s_{k}>0$, it follows that $s_{k+1}=\Delta x_{k}+s_{k}-\phi$. If (5) holds, then $-\varepsilon <s_{k+1}<s_{k}$, where $\varepsilon =\left|\Delta x_{k}\right|+\phi$. Following similar lines, for case-2, where $s_{k}<0$, this leads to $\varepsilon >s_{k+1}>s_{k}$.

The proof of the second point of Theorem 1 can be also divided into two cases as before i.e., when $s_{k}>0$ and $s_{k}<0$. Let us consider case-1 where $s_{k}>0$. The mth iteration of (3) is expressed by

(7) $$\begin{array}{cc} s_{m} & \leq s_{0}+m\left(\chi -\phi \right)\;. \end{array}$$

From the first point of Theorem 1, there exists a $k_{f}$ step where $\delta_{k_{f}}\neq \delta_{k_{f}-1}$. Let us assume the maximum *m* such that Δx(k)=χ, and $s_{\left\lfloor m\right\rfloor }\leq 0$ in (7). It can be seen that the inequality (7) implies

$$m\leq \frac{s_{0}}{\phi -\chi }\;.$$

The same results can be found when $s_{k}<0$ which concludes the proof of the second point Theorem 1.

For a special case of input signal which does not change over time i.e., $\Delta x_{k}=0$, the switching function in (3) becomes

$$\begin{array}{cc} s_{k+1} & =s_{k}-\phi \delta_{k} \end{array}$$

From the first point of Theorem 1, the QSMD becomes $\left[-\phi ,+\phi \right]$ (i.e., the global attractor on $\left(-\infty ,\infty \right)$. Let $k_{f}$ denotes the first step after $s_{k}$ crosses switching manifold such that $\left(\delta_{k_{f}}\neq \delta_{k_{f}-1}\right)$. Next, we will study two possible cases that will take place for the sliding state $s_{k}$ inside QSMD, i.e., $0<s_{k}\leq \phi$ and $-\phi <s_{k}<0$. When $0<s_{k}\leq \phi$, it follows that

(8a) $$\begin{array}{cc} s_{k+1} & =s_{k}-\phi \end{array}$$

(8b) $$\begin{array}{cc} s_{k+2} & =s_{k+1}+\phi \end{array}$$

(8c) $$\begin{array}{c} =s_{k} \end{array}$$

Similarly, when $-\phi <s_{k}<0$ it can easily be shown that $s_{k+2}=s_{k}$. It should be pointed out that once the sliding state $s_{k}$ becomes inside the QSMD defined by $\left|s(k)\right|<\phi$, it will also converge to the periodic-2 orbit.  □

## 3. Adaptive Delta Modulator

The fixed Δ-M is replaced by its adaptive form, i.e., adaptive Δ-M, to increase its region of attraction and reduce the quantization noise. The adaptation scheme in the adaptive Δ-M aims to update the gain of the quantizer (ϕ) according to the variation of the input signals. For example, the parameter ϕ enlarges for high frequency input signals and decreases when the input signal is of low frequency. Thus, to avoid the slope-overload distortion and to increase the attraction area (stability region) of the modulator all the time, the adaptive mechanism inside the Δ-M should have a growth rate higher than the slope of the input signal.

The dynamic of the adaptive Δ-M can be written as

(9a) $$\begin{array}{ccc} {\hat{x}}_{k+1} & = & {\hat{x}}_{k}+\phi_{k}\;\delta_{k} \end{array}$$

(9b) $$\begin{array}{ccc} s_{k} & = & x_{k}-{\hat{x}}_{k} \end{array}$$

(9c) $$\begin{array}{ccc} \phi_{k} & = & \phi_{k-1}\mu_{k},\;\phi_{0}>0 \end{array}$$

where $x_{k}\in R$, ${\hat{x}}_{k}\in R$ denote the input of the encoder $E_{A\Delta }^{x}$, the output of decoder $D_{A\Delta }^{x}$, $\phi_{k}\in R_{+}$ denotes the adaptive gain of the two-level quantizer component and

(10) $$\begin{array}{cc} \mu_{k} & =m+\frac{1}{2}\left(M-m\right)\left|\delta_{k}+\delta_{k-1}\right| \end{array}$$

where 0<m<1 and M>1. Substituting (9b) and (9c) into (9a) gives

(11) $$\begin{array}{cc} {\hat{x}}_{k+1} & =x_{k}-s_{k}+\phi_{k-1}\mu_{k}\;\delta_{k} \end{array}$$

Using (11) and following similar lines in Lemma (1) gives

(12) $$\begin{array}{cc} s_{k+1} & =\Delta x_{k}+s_{k}-\phi_{k-1}\mu_{k}\delta_{k} \end{array}$$

which denotes the switching function of (9).

**Remark** **1.** *The factor $\mu_{k}$ falls under one of the following two cases* ***:***

- ***Case-I:** If $s_{k}$ and $s_{k-1}$ are both positive (negative), then the binary sequence $\delta_{k}=\delta_{k-1}=1$$\left(\delta_{k}=\delta_{k-1}=-1\right)$. This implies $\mu_{k}=M$ which denotes the exponential growth rate of $\phi_{k}$*
- ***Case-II:*** *If $s_{k}$ and $s_{k-1}$ have different signs, then the binary sequence $\delta_{k}\neq \delta_{k-1}$. This implies $\mu_{k}=m$ which denotes the exponential decay rate of $\phi_{k}$*

### 3.1. Stability Analysis for Adaptive Δ-M

The following theorem is devoted to derive the stability conditions of the adaptive Δ-M.

**Theorem** **2.** *Recall the system (9). For any bounded input signal with*

(13) $$\begin{array}{c} \left|x_{k}\right|\leq \gamma \; \end{array}$$

(14) $$\begin{array}{c} \left|\Delta x_{k}\right|\leq \chi \end{array}$$

*and starting from any initial step $s_{0}$, the system satisfies the first and second conditions of QSM within $\left\lfloor h\right\rfloor \in N$ where (  denotes the floor operation). This is defined by*

$$\begin{array}{cc} h & =\log_{M}\left(\frac{\chi }{\phi_{0}}\right) \end{array}$$

*and converges to QSMD $\left|s_{k}\right|\leq \varepsilon$ within $\left\lfloor l\right\rfloor \in N$ steps, where*

(15) $$\begin{array}{cc} \varepsilon & =\chi +\phi_{0}M^{h} \end{array}$$

(16) $$\begin{array}{cc} l & =\log_{M}\left(1-\frac{\left(1-M\right)\left(r-{\hat{x}}_{0}\right)}{\phi_{0}}\right) \end{array}$$

*Once the system state $s_{k}$ enters into the QSMD, it will remain there for rest of the time.*

**Proof.**  Recall the system (9). In the following, we will study the two possible cases when the system state starts outside QSMD, i.e., $s_{0}>0$ and $s_{0}<0$.

**Case 1** (When $s_{0}>0$): Given that $s_{0}>0$, then $\delta_{0}=\delta_{1}=\cdots =\delta_{l-1}=1$ where l≥1 which denotes the change of sign step i.e., $\delta_{l-1}\neq \delta_{l}$ to be estimated later. Substituting (10) into (12) gives

(17a) $$\begin{array}{cc} s_{1} & =\Delta x_{0}+s_{0}-\phi_{0} \end{array}$$

(17b) $$\begin{array}{cc} s_{2} & =\Delta x_{1}+s_{1}-\phi_{0}M \end{array}$$

(17c) $$\begin{array}{c} =x_{2}-{\hat{x}}_{0}-\phi_{0}\left(1+M\right) \end{array}$$

Iterating (17) for *h* times yields

(18a) $$\begin{array}{cc} s_{h+1} & =\Delta x_{h}+s_{h}-\phi_{0}M^{h} \end{array}$$

(18b) $$\begin{array}{c} =x_{h+1}-{\hat{x}}_{0}-\phi_{0}\sum_{n=0}^{h}M^{n} \end{array}$$

Since M>1 and $\left|\Delta x_{k}\right|\leq \chi$, then there exists *h* such that $\left|\Delta x_{k}\right|\leq \chi <\phi_{0}M^{h}$. This leads us to the following inequality $s_{h+1}<s_{h}$ which shows that (12) satisfies the first reaching condition of QSM, i.e., (1a).

**Case 2** (when $s_{0}<0$): By following the same argument, it can easily be verified that the switching function of adaptive Δ-M (12) satisfies second reaching condition of QSM (1b), i.e., $s_{h+1}>s_{h}$ when $s_{0}<0$.

From the above discussion, it can be shown that $\left|s_{k+1}\right|<\left|s_{k}\right|$ outside QSMD. This guarantees the monotonic decrease and increase of $s_{k}$ when $s_{0}>0$ and $s_{0}<0$, respectively. As a result, the trajectory will continually slide to enter QSMD within a finite number of steps. It further shows that there exists a step *l* where $\delta_{l}\neq \delta_{l+1}$. To find the bound of *l*, let us consider the first case when $s_{0}>0$. The *l*th iteration of (18b) is expressed by

(19) $$\begin{array}{cc} s_{l} & =x_{l}-{\hat{x}}_{0}-\phi_{0}\sum_{n=0}^{l-1}M^{n}\\ & \leq \gamma -{\hat{x}}_{0}-\phi_{0}\frac{1-M^{l}}{1-M} \end{array}$$

which satisfies (16).

Let the motion of $s_{k_{f}}$ to QSMD is bounded by $k_{f}$ such that $\left|s_{k_{f}}\right|\leq \varepsilon$ for all $k\leq k_{f}$. It clearly implies that once $s_{k_{f}}$ enters into the QSMD, it can not escape it afterward, that is $k>k_{f}$ as per the third condition of the reaching law for QSM. To prove this part, we have to investigate the two possible cases when system trajectory enters QSMD, i.e., $0\leq s_{k}\leq \varepsilon$ and $-\varepsilon \leq s_{k}<0$.

**Case 1** (When 0≤s(k)≤ε): We can rewrite (18a) as

$$s_{k+1}=\Delta x_{k}+s_{k}-\phi_{0}M^{h}$$

Assume that $\chi +\phi_{0}M^{h}<\varepsilon$. Considering the worst scenario when $\Delta x_{k}=-\chi >-\phi$ and $s_{k}=0$, it can be shown that $s_{k+1}\geq -\varepsilon$.

**Case 2** (When −ε≤s(k)<0): Similarly, it can easily be verified that $s_{k+1}\leq \varepsilon$ when −ε≤s(k)<0. This shows that once the trajectory goes into the QSMD defined by $\left|s_{k}\right|\leq \varepsilon$, it will never escape it. The proof of Theorem is complete by satisfying the third reaching Condition 2.  □

### 3.2. Periodic Orbits for Adaptive Δ-M with dc Input Signal.

This part studies the behavior of adaptive Δ-M with a dc input signal, i.e., $\Delta x_{k}=0$.

**Lemma** **1.** *For a special case of a constant input signal $x_{k}$ whose rate of change is zero, i.e., $\Delta x_{k}=0$, and m=1/M, the trajectory of* (9) *will converge to an a symmetric periodic-4 orbit.*

**Proof.**  For a special case of adaptive Δ-M (9) with the input signal which is constant, i.e., $\Delta x_{k}=0$, and m=1/M the switching function in (12) becomes

$$\begin{array}{cc} s_{k+1} & =s_{k}-\phi_{k}\delta_{k} \end{array}$$

Consider the initial condition with $s_{0}\geq 0$ and assume that $\delta_{-1}=\delta_{0}=\delta_{1}=\cdots =\delta_{l-1}=1$ where l≥1 and *l* denotes the change sign step. It follows that

(20a) $$\begin{array}{cc} \phi_{l-2} & =\phi_{l-3}M=\phi_{0}M^{l-1} \end{array}$$

(20b) $$\begin{array}{cc} \phi_{l-1} & =\phi_{l-2}M=\phi_{0}M^{l} \end{array}$$

and

(21a) $$\begin{array}{cc} s_{l-1} & =s_{l-2}-\phi_{l-2}\\ & =s_{l-2}-\phi_{0}M^{l-1}\geq 0 \end{array}$$

(21b) $$\begin{array}{cc} s_{l} & =s_{l-1}-\phi_{l-1}\\ & =s_{l-1}-\phi_{0}M^{l}<0 \end{array}$$

In other words, there exists *l* such that $\phi_{0}M^{l-1}<s_{l-2}$ and $s_{l}<0<s_{l-1}<\phi_{0}M^{l}$. Given that $\delta_{l}\neq \delta_{l-1}$, it can be shown that $\phi_{l}=\frac{\phi_{l-1}}{M}=\phi_{0}M^{l-1}$ and

(22) $$\begin{array}{cc} s_{l+1} & =s_{l}+\phi_{0}M^{l-1} \end{array}$$

One of the following two cases holds for (22): **Case 1:** $\left|s_{l}\right|\leq \phi_{0}M^{l-1}$ and **Case 2:** $\left|s_{l}\right|>\phi_{0}M^{l-1}$. Let us start with the first case, i.e., $\left|s_{l}\right|\leq \phi_{0}M^{l-1}$. It follows that $s_{l+1}\geq 0$, $\delta_{l+1}\neq \delta_{l}$ and it follows that $\mu_{l+1}=\frac{1}{M}$, accordingly. The next step $s_{l+2}=s_{l+1}-\phi_{0}M^{l-2}$ gives similar results if $\left|s_{l+1}\right|\leq \phi_{0}M^{l-2}$ still holds. Clearly, the adaptive quantizer step-size $\phi_{k}$ will continuously decrease with every iteration as long as $\left|s_{k}\right|\leq \phi_{k}$ holds.

Iterating (22) $l^{*}$ times gives $s_{p}=s_{p-1}-\Psi \delta_{p-1}$ where $p=l+l^{*}$ and $\Psi =\phi_{0}M^{2l-p}$. It is clear that there is a finite number of iteration *p* where (22) switches from case 1 to case 2, i.e., $\left|s_{p-2}\right|\leq \Psi M$ and the following step satisfies $\left|s_{p-1}\right|>\Psi$, which implies $\delta_{p}=\delta_{p-1}$. If $\delta_{p-2}=-1$ then $\delta_{p}=\delta_{p-1}=1$, and

(23a) $$\begin{array}{cc} s_{p-1} & =s_{p-2}+\Psi M \end{array}$$

(23b) $$\begin{array}{cc} s_{p} & =s_{p-1}-\Psi \end{array}$$

(23c) $$\begin{array}{cc} s_{p+1} & =s_{p}-\Psi M\\ & =s_{p-2}-\Psi \end{array}$$

It is easy to show that $\left|s_{p}\right|<\Psi M$ since $\left|s_{p}\right|<\left|s_{p-1}\right|<\Psi M$.

$$\begin{array}{cc} s_{p+2} & =s_{p+1}+\Psi \\ & =s_{p-2} \end{array}$$

Following similar steps gives $s_{p+3}=s_{p-1}$, $s_{p+4}=s_{p}$ and $s_{p+5}=s_{p+1}$, which completes the proof.  □

## 4. Simulation

It is instructive to investigate the dynamical properties of both fixed Δ-M and its adaptive counterpart. Thus, simulation results for both systems using MATLAB/Simulink are presented in this section. The input and output signals in addition to the sliding state inside both systems are shown and their behavior in the possible operating regions are vividly analysed considering the following signal [24]

(24) $$y\left(t\right)=\left\{\begin{array}{c} 3\;r(t)-r(t-5)-2\;r(t-10)\;0\leq t\leq 15\\ \sin(0.7t)+\sin(0.256t)+5.89\sin(0.385t+2.5)\\ \;\;\;\;+26.89\;\;15<t\leq 30 \end{array}\right.$$

where r(t) is a ramp signal. This signal acts as the input of both the fixed and the adaptive Δ-M.

### 4.1. Simulation Results of Fixed Δ-M

**Figure 2:**
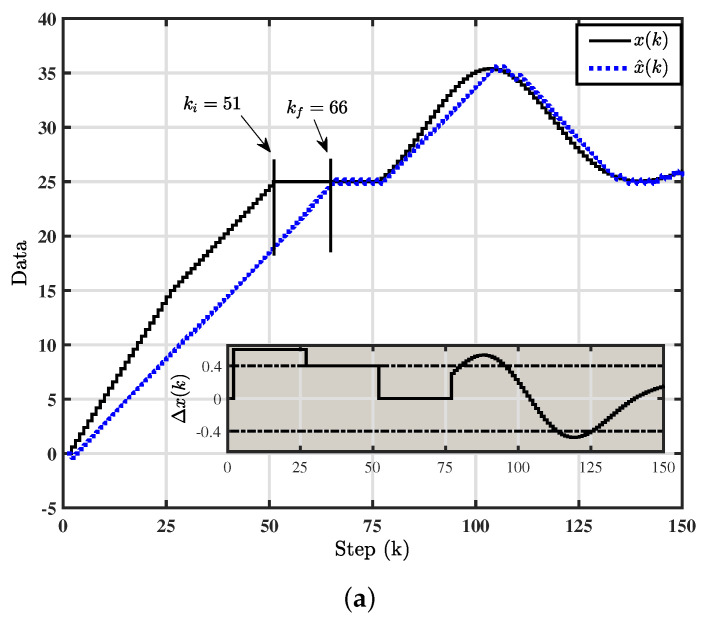

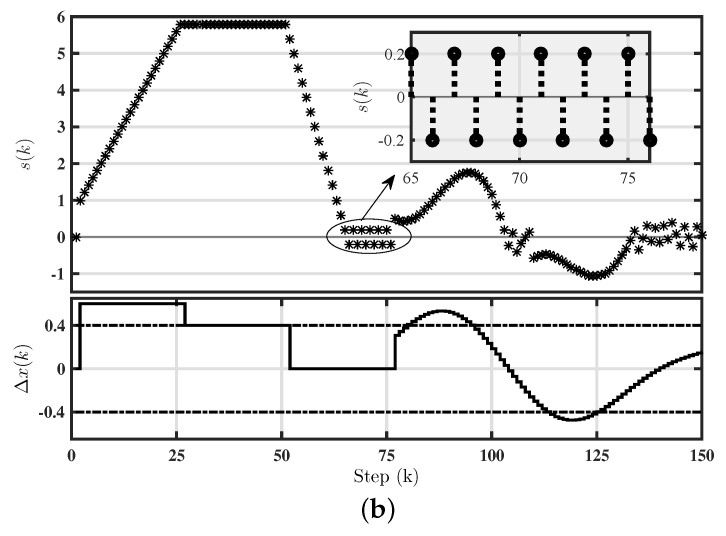
(**a**) The various trajectories of fixed D-M; (**b**) Switching function of fixed Δ-M.

In this work, we consider the discretized equivalent signal of (24) (denoted as y(k)), $\forall t\in \left[hk,(k+1)h\right]$. We then apply y(k) as the input of the fixed Δ-M (2) such that x(k)=y(k). The various trajectories of fixed Δ-M are depicted in Figure 2a for ϕ=0.4, h=0.2 and s(0)=0. As per the stability condition (5), one can observe the converging trajectories which shows that fixed Δ-M is stable for all $\left|\Delta x(k)\right|\leq 0.4$. Next, the output of the fixed Δ-M, namely, $\hat{x}\left(k\right)$ diverges from the system input x(k) with Δx(k)=0.6∀0≤k<25 and it converges again when Δx(k)≤0.4∀50≤k<65; since it satisfies stability conditions. As per (6), the number of steps takes the trajectory takes to move from the marginal mode starting at k=51 to equivalent mode is 15 which yields $k_{f}=66$.

Switching function of fixed Δ-M is shown in Figure 2b. Periodic orbit of fixed Δ-M has been found according to the third point of Theorem 1 where the trajectory of fixed Δ-M converges to a period-2 orbit in the staedy state, i.e., Δx(k)=0 between step number $k_{f}=66$ and 75.

### 4.2. Simulation Results of Adaptive Δ-M

**Figure 3:**
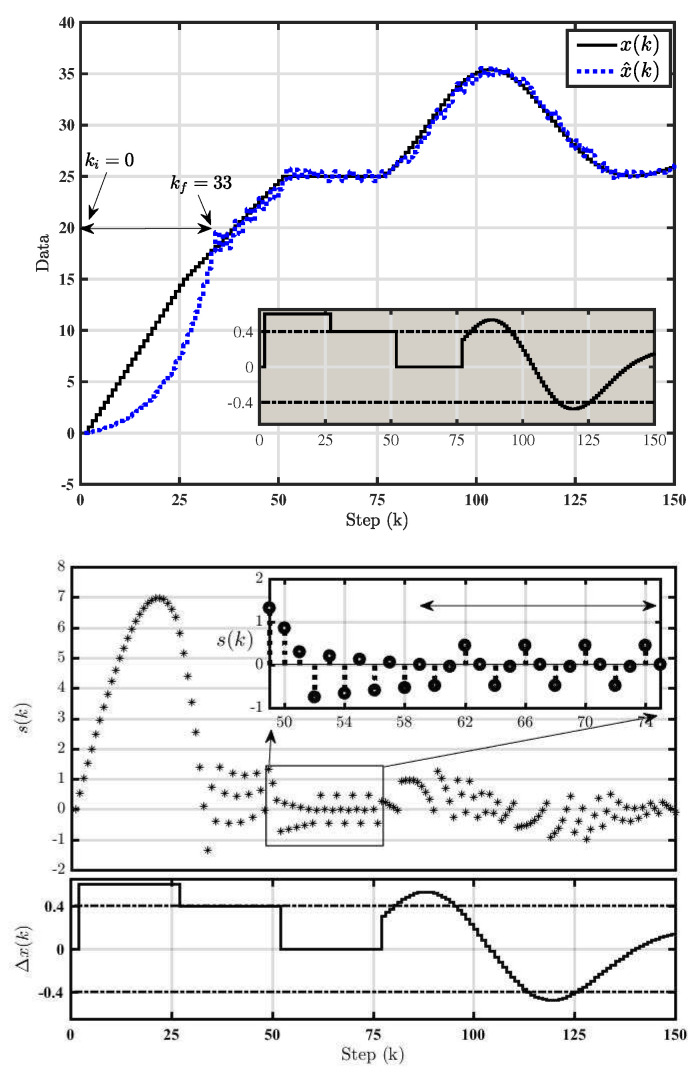
Adaptive Δ-M.

The trajectories of adaptive Δ-M system are shown in Figure 3 for ϕ=0.4, h=0.2, M=1.1, $m=\frac{1}{M}$ and s(0)=0. According to the stability condition (5), it is shown that adaptive Δ-M satisfied QSM conditions from the first step. Moreover, the number of step, which takes the trajectory to move to the equivalent mode, equals 33.

Switching function of adaptive Δ-M is shown in Figure 2b. Periodic orbit of adaptive Δ-M has been found according to Lemma (2), where the trajectory of its adaptive counterpart converges to a period-4 orbit in the steady state, i.e., Δx(k)=0 between step number 59 and 75.

## 5. Conclusions

In this paper, the stability analysis, periodicity and accurate approximation of hitting-step for both data-driven fixed and adaptive Δ-M have been derived and analytically investigated using quasi-sliding mode theory. Some properties of the input signals in addition to the gain of the two-level quantizer in both fixed Δ-M and adaptive Δ-M have been utilized to derive stability conditions and approximate of the hitting-time. Moreover, the existence of periodicity in the steady state of both types of Δ-M, is derived. We found that, with appropriate choices of quantizer gain and parameters of the fixed and adaptive Δ-M, both fixed and adaptive Δ-Mconverge to periodic-2 and periodic-4 orbit, respectively. The findings in simulation section verify the theoretical analysis.

## Abbreviations

The following abbreviations are used in this manuscript:

Δ-M	Delta Modulator
$\Delta_{H}$-M	Hybrid Delta Modulator
ΔΣ-M	Delta Sigma Modulator
ADC	Analogue to Digital Converter
CT	Continuous-Time
DAC	Digital to Analogue Converter
DT	Discrete-Time
NCS	Networked Control System
QSM	Quasi-Sliding Mode
QSMD	Quasi-Sliding Mode Domain
SS	Steady State
TP	Transient Process
